# Urban Sustainability Evaluation under the Modified TOPSIS Based on Grey Relational Analysis

**DOI:** 10.3390/ijerph16020256

**Published:** 2019-01-17

**Authors:** Juan Tang, Hong-lin Zhu, Zhi Liu, Fu Jia, Xiao-xue Zheng

**Affiliations:** 1 College of Management Engineering, Anhui Polytechnic University, Wuhu 241000, China; juan1985juan@163.com; 2 School of Economics and Management, Jiangsu University of Science and Technology, Zhenjiang 212003, China; zhlin96@163.com; 3 York Management School, The University of York, Heslington, York YO10 5DD, UK; fu.jia@exeter.ac.uk; 4 College of Transportation and Civil Engineering, Fujian Agriculture and Forestry University, Fuzhou 350002, China; snowwie@126.com

**Keywords:** urban sustainability, evaluation index system, modified TOPSIS, grey relational analysis

## Abstract

The evaluation of urban sustainability plays a crucial role in the process of the sustainable development of cities. To decrease subjectivity and attain a comprehensive evaluation, this paper develops an evaluation method using the technique for order preference by similarity to ideal solution (TOPSIS). First, an evaluation index system including 39 indices and three categories (economic, social, and ecological development) is established; second, based on the index system, a modified TOPSIS, in which the entropy method is used to assign weights to each index according to its evaluation score and grey relation analysis is used to reduce the uncertainty existing in the process of evaluation, is presented to rank the sustainability level of cities. Finally, an example with the sustainability evaluation of 16 cities in the Anhui province of China is introduced to verify the effectiveness of the model.

## 1. Introduction

The development of human society is facing many challenges, including an exploding population, inadequate or failing infrastructure, inequalities among regional economies, resource shortages, and environmental disruptions [1,2]. These problems have become vital factors restricting the progress and development of human society. To attain the coordinated development of economic, social, resource, and environmental protection, sustainable development was first proposed in the World Conservation Strategy, which was jointly published by the International Union for Conservation of Nature (IUCN), the United Nations Environment Programme (UNEP), and the World Wildlife Fund (WWF) in 1980 [3]. Then, many definitions of sustainable development appeared, and the most popular definition was provided by the Brundtland Report in 1987: “Development that meets the needs of the present without compromising the ability of future generations to meet their own needs”. [4]. Sustainable development is also a process in which resource utilization, investment orientation, technological development, and policy changes are coordinated in order to continuously promote the potential to meet human needs both now and in the future [1]. In this process, development is the core, but it requires economic and social development under strict control of population, the improvement of population quality, the protection of the environment, and the sustainable utilization of resources.

As a carrier of human habitation, the city is closely related to people’s daily life. Currently, more than 50% of the world’s population lives in cities [5]. Moreover, cities in the developing world will account for 95% of urban growth and be home to almost 4 billion people by 2030 [6]. In addition, the world population is projected to be 70% urban by 2050 [7]. Thus, as the main constituent of nations and the world, cities play an important role in promoting human social and economic activities in a concentrated manner [6], and its sustainable construction is the key of nations’ and the world’s sustainable development [8]. In September 2015, the UN adopted 17 sustainable development goals. Among them, Goal 11 is “to build an inclusive, safe, and resilient sustainable city and human settlements” [9]. It can be seen that in the context of the rapid development of urbanization, urban sustainable development has become the focus in various countries and regions of the world.

Sustainability is a series of conditions or ideal statuses, which is the goal of sustainable development [10]. As one extension of sustainability, urban sustainability is the conditions or ideal status of a particular period in the process of urban sustainable development, and it involves economic, social, and environmental aspects [11,12]. In the process of urban sustainable construction, the stakeholders and policy-makers need to resort evaluation indices and method to assess the status of urban sustainable development and allow for comparison against other cities, then make them recognize the strengths and weaknesses of urban development from perspectives of society, economics, and environment after one period, which supports them to make the development planning and correct policy interventions to guarantee the goal of urban sustainable development [13,14,15]. Therefore, the evaluation of urban sustainability is one crucial step and plays a key role in the process of urban sustainable development. In theory and practice, the evaluation of urban sustainability has also drawn significant attention and has been extensively studied [16,17,18,19,20,21,22,23,24,25,26,27,28,29]. However, among these studies, most of indices are repetitive in the classification of the index system of urban sustainability. In addition, when using a mathematical model for evaluation, only some certain dimensions are considered rather than considering the comprehensiveness of the influencing factors. Moreover, the current research is still insufficient for the evaluation of urban sustainability using multi-attribute decision-making methods, and many evaluation methods have certain limitations. For example, the analytic hierarchy process (AHP) is influenced by subjective factors, and elimination et choix tradulsant la realitite (ELECTRE) and preference ranking organization method for enrichment evaluation (PROMETHEE) do not need the process of nondimensionalization, which often leads to the lack of information and results. In this paper, we use the objective data of the statistical yearbook and an improved approach to determine the index weight and evaluate urban sustainability, which can reduce subjectivity as much as possible in the evaluation process.

In this paper, we are committed to exploring a way to objectively evaluate urban sustainability. We first conduct a literature review about the definition and the evaluation approach of urban sustainability in Section 2. Section 3 constructs an evaluation index system about urban sustainability. In Section 4, we introduce the approach of evaluating urban sustainability. Section 5 uses the evaluation approach for a case study of the Anhui Province in China. Conclusions and further studies are drawn in Section 6.

## 2. Literature Review

### 2.1. The Definition of Urban Sustainability

Based on the Brundtland Report, many scholars have given an improved interpretation of sustainability to elaborate on the relation between nature and humans and their generation [30,31,32]; however, the definition of urban sustainability is still vague, and sustainability at the urban level has special characteristics [33]. The concept of sustainability has a significant influence on planning and policy at the local level [9]. Therefore, the definition of urban sustainability is important for the sustainable evaluation.

Urban development relies on the external environment for its resource inputs, and it also needs to export waste to the external environment [34]. Thus, Daly and Cobb [17] define urban sustainability from four aspects: (1) The environmental impact of urban industrial development and human activities should be less than the environmental carrying capacity of the city; (2) the consumption rate of renewable resources in social production and human activities should be less than the rate of regeneration; (3) the discharge amount and rate of all kinds of wastes in social production and human activities should be within the scope of the urban environmental self-purification capacity; and (4) the consumption rate of nonrenewable resources in social production and human activities should be less than that of other renewable resources. Based on the definition of sustainability, Hamilton et al. [18] propose that urban sustainability is “the process of developing a built environment that meets people’s needs whilst avoiding unacceptable social or environmental impacts”. Chi et al. [19] state that a sustainable city is one that relates its use of resources and its generation and disposal of wastes to the limits imposed on such activities by the planet and its organisms. Zhou et al. [20] consider that a sustainable city is a complicated engineering system that involves many responsible departments assuming various functions, such as political, economic, environmental, cultural, and others. Mori and Yamashita [5] state that city sustainability denotes the maximization of economic and social net benefits under the limitations of environmental burdens and within the acceptable limits of economic and social inequity. Tursun et al. [8] consider that urban sustainability is based on social sustainability, economic sustainability, and environmental sustainability. Social sustainability is the goal, environmental sustainability is the foundation, and economic sustainability is the condition. In addition, Li et al. [35] propose that the essence of urban sustainability is the necessary conditions for a series of cities, an idealized urban state, and the development goal of most cities. Wu [21] considers that sustainability is an adaptive process of facilitating and maintaining a virtual cycle between ecosystem services and human wellbeing through concerted ecological, economic, and social actions in response to changes within and beyond the urban landscape. Based on the concept of the triple bottom line, some four-dimensional frameworks were proposed, such as “society-economy-environment-institution” and “ecology-economy-politics-culture” [36,37]. 

Previous studies on the concept of urban sustainability have shown that urban sustainability has some evident characteristics. First, urban sustainability covers social, economic, and ecological factors, and the corresponding subsystems interact with each other. Second, urban sustainability includes the relationship between the city and the external environment. The external environment is a set of external factors (the factors are not inside the urban system and uncontrolled) of urban system, which encompasses national policies, migrant population, the level and development trend of science and technology, and the sustainability of surrounding areas and even the world, and has a compressive impact on the city’s sustainability. Third, a sustainable city is one that succeeds in balancing economic, environmental and sociocultural progress through processes of active citizen participation. These characteristics of urban sustainability indicate that the three subsystems should be coordinated and balanced with each other, thus achieving the stabilization and improvement of the urban system in the process of urban sustainable development. Thus, we can conclude that the conception of urban sustainability is as follows; the state that enables the urban composite system to continuously develop in a balanced, stable, and coordinated direction with comprehensive consideration of the social, economic, and ecological factors.

### 2.2. The Evaluation of Urban Sustainability

Various frameworks and tools have been developed to address the evaluation of urban sustainability, such as the Comprehensive Assessment System for Building Environmental Efficiency (CASBEE), the Leadership in Energy and Environmental Design (LEED), and the British Research Establishment Environmental Assessment Method (BREEAM) [38,39]. Although these tools have been applied in some developed and developing countries their applicability is questioned. To be in line with the local context, scholars have conducted many studies on the evaluation index system and evaluation method, which are two key issues of urban sustainability evaluation. As for the evaluation index system for urban sustainability, Huang et al. [40] discuss the ecological economic dimensions of urban sustainability, and 80 indices are selected through the participation of nongovernmental organizations, which can be used as policy-making indices for measuring Taipei’s urban sustainability. Kerk and Manuel [41] propose a highly aggregated sustainability index, the Sustainable Society Index (SSI), which integrates the most important aspects of the sustainability and life quality in a simple and transparent way. It consists of only 22 indices that are grouped into five categories. Ding et al. [42] propose an inclusive, causal framework for sustainable development assessments entitled the “Trinity of Cities’ Sustainability from Spatial, Logical, and Time Dimensions” (TCS-SLTD). Shen et al. [43] selected five different sets of indices and form a list named the international urban sustainability indices, which is used to develop a comparative basis for analyzing how different practices comply with its environmental, economic, social, and governance indices. In addition, some other indices are proposed, such as Genuine Savings (GS), the Genuine Progress Indicator (GPI), Sustainable Transportation Indicators (STI), and so forth [44,45,46]. These system indices include three dimensions (the economy, society, and ecology) of urban sustainability, but some other indices only consider one or two dimensions. For example, the Environmental Sustainability Index (ESI) and the Environmental Performance Index (EPI) are the indices that only consider social and ecological dimensions. They were developed by Columbia University and Yale University in 2005 and 2006, respectively [47,48,49]. The ESI aggregates 76 variables into 21 indices, resulting in five categories. The EPI comprises six categories (Environmental Health, Biodiversity and Habitat, Sustainable Energy, Water Resources, Air Quality, and Productive Resource Management), which are derived from 16 indices. The Ecological Footprint (EF) proposed by Rees focuses on ecological sustainability by using area-based indicators with the aim to make the city operate within its carrying capacity [50]. However, this method has some obvious disadvantages. It ignores the sustainability of social and economic systems, and it does not consider the impacts of external factors on the urban system. In addition, most carrying capacity evaluation methods assume that the supply of urban resources does not change, but in fact human activities will have a certain impact on the supply of resources, which is likely to lead to erroneous results.

Extensive research has been conducted for the evaluation method of urban sustainability based on an index system. For example, Rajaonson and Tanguay [51] apply the linear aggregation and the Borda rule to estimate the overall sustainability score of 25 cities in Quebec. Li et al. [13] developed a Full Permutation Polygon Synthetic Indicator method to evaluate the capacity for urban sustainable development at different times during the next two decades. Haghshenas et al. [52] analyzed the environmental, economic, and social sustainability of urban transportation to investigate the impact of various transportation policies using system dynamics. Hély et al. [53] applied fuzzy logic for evaluating the value of each indicator, and arithmetic and geometric means for aggregated assessment of sustainability from economic, social, and environmental spheres. Mahmoud et al. [39] developed a globally sustainability rating tool for existing building based on a fuzzy multicriteria decision-making method. Ameen and Mourshed [38] took Iraq as a case study and developed a stakeholder-driven structured methodology, which identifies and ranks context-relevant indicators and assigns weights for aggregating indicator scores by applying analytic hierarchy process. 

Among these evaluation methods, multicriteria decision methods (MCDMs) have been demonstrated as effective indicator system evaluation methods in the literature [38,54,55,56]. The most commonly used MCDM methods are the Analytic Hierarchy Process (AHP), ELECTRE, PROMETHEE, and NAIADE [56,57,58,59]. MCDMs can make evaluation process become inter-/multidisciplinary (with respect to the research team), participatory (with respect to the local community), and transparent (since all criteria are presented in their original form without any transformations in money, energy, or another common measurement method), which accomplishes not only the sustainability evaluation of one city, but also the comparison of development level among different cities. In addition, it is also appropriate to evaluate the performance of sustainability policies. However, the interactions of different cities and of different subsystems in one city subsystems can reduce the accuracy of MCDMs in comparison of sustainability level among different cities. Therefore, we need another method to measure and compare the sustainability of different cities. In this paper, we use the technique for order of preference by similarity to ideal solution (TOPSIS) with grey relational analysis. TOPSIS can obtain the positive and the negative ideal solutions of urban sustainability through the data of each city’s indicators, and these ideal solutions provide a standard for the comparison of the sustainable development level of each city. By comparing the degree of differentiation between the ideal solutions and the data of cities, the disparity in the urban sustainability can be acquired. Then, the related agents have certain cognition of the sustainable development statuses of cities, which allows them to further understand the strengths and weaknesses of urban sustainable development. Grey relational analysis is a quantitative method to analyze the correlation degree among various factors in the grey system, and it is helpful to reducing the subjectivity that is generated in the process of setting the criteria. Therefore, TOPSIS combined with grey relational analysis can effectively evaluate the differentiation of the sustainable development levels among cities.

## 3. Urban Sustainability Evaluation Index System

Urban sustainability needs to be evaluated using an all-directional and multiangled approach. From the definition of urban sustainability, this all-directional and multiangled evaluation can assess and monitor the sustainable development level of economy, society, and ecology, then help to prompt continuous, stable, and balanced development of cities. Therefore, one effective urban sustainability evaluation index system should be constructed considering these factors and complying with certain principles so that the evaluation index system is complete, simple, and accurate. For example, the evaluation indices reflect the current situation and future development trends; focus on human activities; and are typical, recapitulative, and accurately defined.

Based on the literature [23,37,60], this paper builds one evaluation index system for urban sustainability and it is shown in Table 1. This system contains 39 indices, and they are divided into three parts: economic indices, social indices, and ecological indices, which are marked as EC, SC, and EL, respectively. In Table 1, the natural growth rate of the population, population density, urban Engel coefficient, rural Engel coefficient, urban unemployment rate, number of criminal cases, total amount of industrial waste gas discharge, total amount of industrial wastewater discharge, yield of industrial solid waste, and urban sewage discharge are cost indices (these indices are expected as small as possible, a smaller value denotes the sustainability level of cities is higher), and the rest are benefit indices (these indices are expected as large as possible, a larger value denotes the sustainability level of cities is higher).

### 3.1. Economic Indices

Economic indices reflect the economic development of the city, which can be measured from the quantity, quality, and efficiency of economic development. In terms of economic development quantity, GDP, investments in fixed assets, the retail sales of consumer goods, and the total import and export volume can be used to reflect the economic scale and economic strength of a city. In terms of economic development quality, GDP per capita, the disposable income of urban residents per capita, the disposable income of rural residents per capita, and the output value of high-tech industries are used to evaluate the effectiveness of the economic structure and development mode. The economic increment is used to analyze the growth rate of aggregate data from the perspective of efficiency and to evaluate the economic development potential of cities, including the GDP growth rate, the growth rate of total investments for fixed assets, the growth rate of consumer goods’ retail sales, and the growth rate of the total import and export volume.

### 3.2. Social Indices

The social system largely reflects the status and the quality of life, science, education, and culture of the entire city. Philosophically, people are the sum of their social relations, and the relationship between people forms the framework of the social system. Therefore, the establishment of social indices should focus on people. The population indices reflect the growth and scale of the urban population and the population capacity, including the natural growth rate of the population and population density. Urban construction is closely related to human activities. For sustainable development, such indices need to reflect peoples’ suitability, comfort, and contributions to optimizing the industrial structure in urban activities, and the indices include the urban Engel coefficient, the rural Engel coefficient, the urban unemployment rate, the percentage of urban residents with basic medical insurance, the number of practicing physicians per 10,000 people, and the number of criminal cases. The science, education, and culture indices can reflect the current educational level and cultural atmosphere of the city and evaluate the city’s innovative potential and future development trends, including the numbers of library collections and patent applications per million people, education years per capita, and the number of graduates from ordinary colleges and universities.

### 3.3. Ecological Indices

The ecosystem presents the urban ecological level and the living environment under the influence of the economic and social system; it is a vital factor of urban sustainability as well.

The evaluation of ecosystems includes three parts: natural resources, environmental pollution, and ecological construction. Natural resources are the material and spatial basis for human survival and development. Such indices reflect the current environmental basic status of urban system, the available quantity of natural resources has important impacts on the sustainable development of the city at present and even in the future. The indices include water resources per capita, agricultural land area, and the forest coverage rate. Environmental pollution reflects the negative impacts of social and economic systems on the ecosystem. It is a critical factor that blocks the sustainable development. The indices include the total amount of industrial waste gas discharge, the total amount of industrial waste water discharge, the amount of industrial solid waste, urban sewage discharge, and the proportion of air quality equal to and better than level II. Ecological construction aims to reduce pollution to the ecosystem and maintain the sustainability of the urban system, and thus it reflects the improvement measures of the ecosystem. These indices include the treatment rate of industrial effluents, the financial expenditures on energy conservation and environmental protection, the green coverage rate of built-up areas, and the comprehensive utilization rate of industrial solid wastes.

The established index system covers the demands and objectives of urban sustainable development in terms of economy, society, and ecology. In this paper, considering the characteristics of the established evaluation system, the TOPSIS based on grey relational analysis is used to evaluate urban sustainability.

## 4. The Evaluation Method of TOPSIS Based on Grey Relational Analysis

The evaluation of urban sustainability depends on the established evaluation indices and the corresponding data derived from the statistical yearbooks of each city. However, due to the subjectivity of decision-makers and objective reasons such as the imperfections in the data in the statistical yearbook, there is a certain degree of uncertainty in the evaluation process, which affects the evaluation accuracy of urban sustainability.

**Figure 1:**
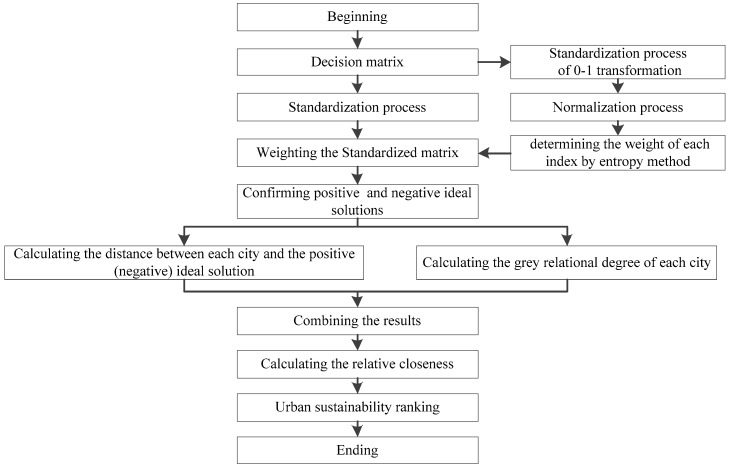
Evaluation process based on the improved the technique for order preference by similarity to ideal solution (TOPSIS).

In view of the uncertainty existing in the system, an improved TOPSIS, in which the entropy method is used to assign weights to each index based on its evaluation score and the grey relation analysis is used to reduce the uncertainty, is applied to assess the numerical values. This improved method can enhance the objectivity of the evaluation, and the specific evaluation process is shown in Figure 1.

### 4.1. The Index Weight Determining Method—Entropy Method

Information entropy can measure the amount of useful information with the data provided. It is an objective way for weight determination when several interrelated objects are evaluated at the same time [61]. In our paper, we need to evaluate the sustainability of multiple cities, and there is close relation among cities. Hence, we choose this method to determine the weight of indices.

The specific steps of this method are as follows. 

**Step 1**: This evaluation system has n indices $\left(G_{1,},G_{2,},\cdots ,G_{n}\right)$ and m cities $\left(A_{1,},A_{2,},\cdots ,A_{m}\right)$. Let $A={\left(a_{ij}\right)}_{m\times n}$ be the decision matrix and $a_{ij}(1\leq i\leq m,1\leq j\leq n)$ be the index value.

(1) $$A=\left[\begin{array}{cccc} a_{11} & a_{12} & \cdots & a_{1n}\\ a_{21} & a_{22} & \cdots & a_{2n}\\ \vdots & \vdots & \cdots & \vdots \\ a_{m1} & a_{m2} & \cdots & a_{mn} \end{array}\right]$$

**Step 2**: Standardize the decision matrix $A={\left(a_{ij}\right)}_{m\times n}$ according to the 0–1 transformation, and get the standardized matrix $D={\left(d_{ij}\right)}_{m\times n}$.

(2) $$\mathrm{Benefit}\;\mathrm{indices}\;\{\begin{array}{l} d_{ij}=\frac{a_{ij}-\underset{i}{\min}\;a_{ij}}{\underset{i}{\max}\;a_{ij}-\underset{i}{\min}\;a_{ij}}\;\underset{i}{\max}\;a_{ij}\neq \underset{i}{\min}\;a_{ij}\\ \;d_{ij}=1\;\underset{i}{\max}\;a_{ij}=\underset{i}{\min}\;a_{ij} \end{array}$$

(3) $$\mathrm{Cos}t\;\mathrm{indices}\;\{\begin{array}{l} d_{ij}=\frac{\underset{i}{\max}\;a_{ij}-a_{ij}}{\underset{i}{\max}\;a_{ij}-\underset{i}{\min}\;a_{ij}}\;\underset{i}{\max}\;a_{ij}\neq \underset{i}{\min}\;a_{ij}\\ \;d_{ij}=1\;\underset{i}{\max}\;a_{ij}=\underset{i}{\min}\;a_{ij}\; \end{array}$$

**Step 3**: Normalization process. Let $Y={\left(y_{ij}\right)}_{m\times n}$ be the normalized matrix.

(4) $$y_{ij}=\frac{d_{ij}}{\sum_{i=1}^{m}d_{ij}}$$

**Step 4**: Calculate the entropy value $E_{j}$ of each index.

(5) $$E_{j}=-\frac{1}{\ln m}\sum_{i=1}^{m}y_{ij}\ln y_{ij}$$

When $y_{ij}=0$, $y_{ij}\ln y_{ij}=0$.

**Step 5**: Calculate the coefficient of variation $h_{j}$ of each index. For one index, the larger that the coefficient of variation h, the smaller that $E_{j}$, the greater the impact of the index on urban sustainability, and the larger the corresponding weight coefficient. Conversely, the smaller that the coefficient of variation h, the larger that $E_{j}$, the lower the impact of the index, and the smaller the weight coefficient [35]. The formula for calculating the coefficient of variation $h_{j}$ is as follows.

(6) $$h_{j}=1-E_{j}$$

**Step 6**: Determine the weight vector $\omega =(\omega_{1},\omega_{2},\ldots ,\omega_{j},\ldots ,\omega_{n})$, where $\omega_{j}$ is determined as follows.

(7) $$\omega_{j}=\frac{h_{j}}{\sum_{j=1}^{n}h_{j}}$$

### 4.2. TOPSIS Incorporating Grey Relational Analysis

The basic idea of TOPSIS incorporating grey relational analysis is as follows. First, one determines the positive ideal solution and negative ideal solution of urban sustainability through the traditional TOPSIS. Then, one uses the grey relational analysis to compare the scores of the evaluation indices of each city with the positive and negative ideal solutions, respectively. Third, the grey relational degree between cities is determined. According to this result, the sustainability of each city is ranked. The specific steps are as follows.

**Step 1**: Standardize the decision matrix $A={\left(a_{ij}\right)}_{m\times n}$ in Equation (1) to obtain a standardized matrix $X={\left(x_{ij}\right)}_{m\times n}$, where $x_{ij}$ is as follows.

(8) $$x_{ij}=\frac{d_{ij}}{\sqrt{\sum_{i=1}^{m}{d_{ij}}^{2}}},\;1\leq i\leq m,\;1\leq j\leq n$$

**Step 2**: Multiply the weight vector $\omega =(\omega_{1},\omega_{2},\ldots ,\omega_{j},\ldots ,\omega_{n})$ obtained in Equation (6) by the standardization matrix $X={\left(x_{ij}\right)}_{m\times n}$ to obtain a weighted standardization matrix as follows.

(9) $$R=\left[\begin{array}{cccc} \omega_{1}x_{11} & \omega_{2}x_{12} & \cdots & \omega_{n}x_{1n}\\ \omega_{1}x_{21} & \omega_{2}x_{22} & \cdots & \omega_{n}x_{2n}\\ \vdots & \vdots & \cdots & \vdots \\ \omega_{1}x_{m1} & \omega_{2}x_{m2} & \cdots & \omega_{n}x_{mn} \end{array}\right]=\left[\begin{array}{cccc} r_{11} & r_{12} & \cdots & r_{1n}\\ r_{21} & r_{22} & \cdots & r_{2n}\\ \vdots & \vdots & \cdots & \vdots \\ r_{m1} & r_{m2} & \cdots & r_{mn} \end{array}\right]$$

**Step 3**: Determine the positive ideal solution $r^{+}$ and the negative ideal solution $r^{-}$ as follows.

(10) $$r^{+}=\left\{\left(\underset{1\leq i\leq m}{\max}r_{ij}|j\in J^{+}\right),\left(\underset{1\leq i\leq m}{\min}r_{ij}|j\in J^{-}\right)\right\}=\left({r_{1}}^{+},{r_{2}}^{+},\cdots ,{r_{n}}^{+}\right)$$

(11) $$r^{-}=\left\{\left(\underset{1\leq i\leq m}{\min}r_{ij}|j\in J^{+}\right),\left(\underset{1\leq i\leq m}{\max}r_{ij}|j\in J^{-}\right)\right\}=\left({r_{1}}^{-},{r_{2}}^{-},\cdots ,{r_{n}}^{-}\right)$$

where $J^{+}$ is the set of benefit indices and $J^{-}$ is the set of cost indices.

The positive ideal solution is the best plan that is a fictitious plan constructed by Equation (10); it means a fictitious city whose sustainability is the best. The negative ideal solution is the worst plan that is a fictitious plan constructed by Equation (11); it means a fictitious city whose sustainability is the worst.

**Step 4**: Calculate the distance between each city and the positive ideal solution $l_{i}^{+}$ and its negative ideal solution $l_{i}^{-}$ as follows.

(12) $$l_{i}^{+}=\sqrt{{\sum_{j=1}^{n}\left[\left(r_{ij}-r_{j}^{+}\right)\right]}^{2}},1\leq i\leq m,1\leq j\leq n$$

(13) $$l_{i}^{-}=\sqrt{{\sum_{j=1}^{n}\left[\left(r_{ij}-r_{j}^{-}\right)\right]}^{2}},1\leq i\leq m,1\leq j\leq n$$

where, $l_{i}^{+}$($l_{i}^{-}$) is the Euclidean distance, a lower $l_{i}^{+}$($l_{i}^{-}$) indicates that the city is closer to the positive (negative) ideal solution; the sustainability level of the city is higher (lower).

**Step 5**: Calculate the grey relational coefficient between the index j of city i and the positive and negative ideal solutions as follows.

The grey relational coefficient with the positive ideal solution is calculated as

(14) $$q_{ij}^{+}=\frac{\underset{i}{\min}\;\underset{j}{\min}\left|r^{+}-r_{ij}\right|+\xi \;\underset{i}{\max}\;\underset{j}{\max}\left|r^{+}-r_{ij}\right|}{\left|r^{+}-r_{ij}\right|+\xi \;\underset{i}{\max}\;\underset{j}{\max}\left|r^{+}-r_{ij}\right|}$$

The grey relational coefficient with the negative ideal solution is calculated as

(15) $$q_{ij}^{-}=\frac{\underset{i}{\min}\;\underset{j}{\min}\left|r^{-}-r_{ij}\right|+\xi \;\underset{i}{\max}\;\underset{j}{\max}\left|r^{-}-r_{ij}\right|}{\left|r^{-}-r_{ij}\right|+\xi \;\underset{i}{\max}\;\underset{j}{\max}\left|r^{-}-r_{ij}\right|}$$

Here, ξ is the distinguishing coefficient, ξ∈[0,1]. ξ=0.5 is normally applied following the rule of least information [62]. Then, the grey relational coefficient matrices $Q^{+}={\left(q_{ij}^{+}\right)}_{m\times n}$ and $Q^{-}={\left(q_{ij}^{-}\right)}_{m\times n}$ are obtained, respectively.

**Step 6**: Calculate the grey relational degree between city i and the positive and negative ideal solutions.

The grey relational degree with the positive ideal solution is

(16) $$q_{i}^{+}=\frac{1}{n}\sum_{j=1}^{n}q_{ij}^{+},\;1\leq i\leq m$$

The grey relational degree with the negative ideal solution is

(17) $$q_{i}^{-}=\frac{1}{n}\sum_{j=1}^{n}q_{ij}^{-},\;1\leq i\leq m$$

**Step 7**: Perform dimensionless processing of the distances $l_{i}^{+}$ and $l_{i}^{-}$, the grey relational degrees ${q_{i}}^{+}$ and ${q_{i}}^{-}$.

(18) $$L_{i}^{+}=\frac{\max{l_{i}}^{+}-{l_{i}}^{+}}{\max{l_{i}}^{+}-\min l_{i}^{+}}\;,\;1\leq i\leq m$$

(19) $$L_{i}^{-}=\frac{\max{l_{i}}^{-}-{l_{i}}^{-}}{\max{l_{i}}^{-}-\min l_{i}^{-}}\;,\;1\leq i\leq m$$

(20) $$Q_{i}^{+}=\frac{{q_{i}}^{+}-\min q_{i}^{+}}{\max{q_{i}}^{+}-\min q_{i}^{+}}\;,\;1\leq i\leq m$$

(21) $$Q_{i}^{-}=\frac{{q_{i}}^{-}-\min q_{i}^{-}}{\max{q_{i}}^{-}-\min q_{i}^{-}}\;,\;1\leq i\leq m$$

where, $L_{i}^{+}$($L_{i}^{-}$) is the dimensionless indicator of the Euclidean distance $l_{i}^{+}$($l_{i}^{-}$), but decreases in $l_{i}^{+}$($l_{i}^{-}$). A higher $L_{i}^{+}$($L_{i}^{-}$) indicates that the city is closer to the positive (negative) ideal solution, the sustainability level of the city is higher (lower). Similarly, a higher $Q_{i}^{+}$ ($Q_{i}^{-}$) has the same meaning.

**Step 8**: Integrate the results of the dimensionless distance and the dimensionless grey relational degree.

(22) $$S_{i}^{+}=\alpha L_{i}^{+}+\beta Q_{i}^{+}\;,\;1\leq i\leq m$$

(23) $$S_{i}^{-}=\alpha L_{i}^{-}+\beta Q_{i}^{-}\;,\;1\leq i\leq m$$

where $S_{i}^{+}$ ($S_{i}^{-}$) denotes the comprehensive relation between city *A_i_* and the positive (negative) ideal solution. α+β=1,α>0,β>0. α and β represent the evaluator’s preference for the curve position and shape, respectively.

**Step 9**: Calculate the relative closeness $c_{i}^{+}$ of city $A_{i}=(i=1,2,\cdots ,m)$

(24) $$c_{i}^{+}=\frac{S_{i}^{+}}{S_{i}^{-}+S_{i}^{+}}\;,\;1\leq i\leq m$$

A higher relative closeness $c_{i}^{+}$ implies that city $A_{i}$ is closer to the positive ideal solution and farther away the negative ideal solution. It means that the sustainability level of $A_{i}$ is higher. In addition, each city can be compared with the ideal solutions to acquire its relative closeness, so this indicator can be used to rank the sustainability level of cities.

**Step 10**: Rank the results according to the relative closeness $c_{i}^{+}$. The larger $c_{i}^{+}$, the closer it is to the positive ideal solution, and the higher the sustainability level of the city.

## 5. Application Research

**Figure 2:**
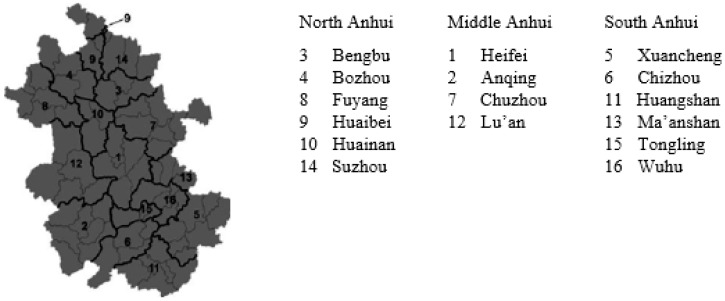
Urban distribution map of Anhui Province.

In this section, 16 cities in Anhui Province are taken as examples to conduct an urban sustainability evaluation and to further confirm the effectiveness of the evaluation method based on the “economic-social-ecological” framework. Based on various indices’ data of each city, the current situation of urban sustainable development in Anhui province is studied, which can provide a reference for the sustainable construction of each city. The urban distribution is shown in Figure 2.

### 5.1. Statistics of the Evaluation Indices of Cities in Anhui Province

The 2017 China City Statistical Yearbook, the 2017 Anhui Statistical Yearbook, and the 2017 Statistical Yearbooks of the 16 cities are used as the data sources (If the results of the statistical data are inconsistent, the 2017 China City Statistical Yearbook and the 2017 Anhui Statistical Yearbook shall prevail in turn). Through the collection and processing of the relevant data, the index data of the 16 cities in Anhui province are derived and shown in Table 2 and Table 3.

### 5.2. Numeral Calculations

The sustainability of the 16 cities in Anhui province is evaluated using the mathematical model established in this paper.

#### 5.2.1. Calculating the Weight of Each Index

According to Equations (1)–(4), the decision matrix A is established, standardized, and normalized, and then the entropy value *E_j_* and weight $\omega_{j}$ of each index are calculated by using Equations (5)–(7). The specific calculation results are shown in Table 4.

#### 5.2.2. Calculating the Positive and Negative Ideal Solutions

According to Equations (8) and (9), the decision matrix A is standardized and weighted, then the positive and negative ideal solutions of each index are calculated by using Equations (10) and (11). The results are shown in Table 5.

#### 5.2.3. Calculating the Distance from the Sustainability of Each City to the Positive and Negative Ideal Solutions

According to Equations (12), (13), (18) and (19), the distance between the sustainability of each city and the positive (negative) ideal solution are calculated and nondimensionalized. The results are shown in Table 6.

#### 5.2.4. Calculating the Grey Relational Degree of Each City

Based on the data in Table 5, one calculates the grey relational degree of each city according to Equations (14)–(17), and then nondimensionalizes them using Equations (20) and (21). The results are shown in Table 7.

#### 5.2.5. Calculating the Relative Closeness and Ranking Each City

Based on Table 6 and Table 7, ${S_{i}}^{+}$ and ${S_{i}}^{-}$ are obtained according to Equations (22) and (23), then the relative closeness of each city is calculated using Equations (24), and the sustainability level of each city can be ranked. The results are shown in Table 8.

### 5.3. Comparative Analysis

**Figure 3:**
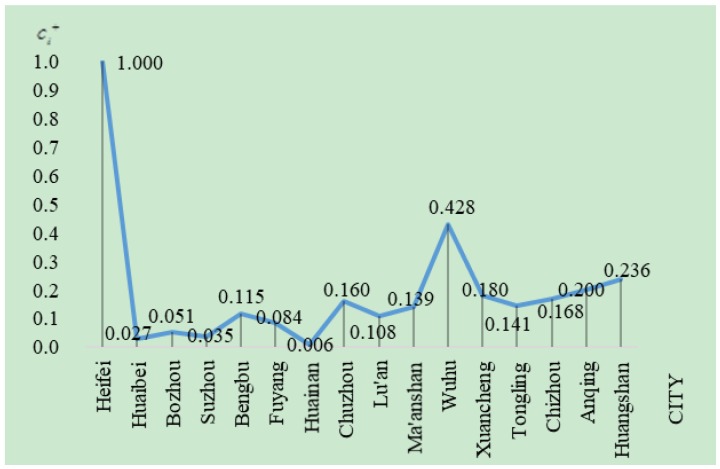
The comparison of sustainability of cities in Anhui Province in 2016.

Figure 3 is drawn according to the results in Table 8. It intuitively shows the sustainability level of each city in Anhui province. From a city perspective, Hefei has the highest scores in Anhui province, namely, the city has the highest sustainability level. The sustainability level is the second highest in Wuhu and the lowest in Huainan. From a regional perspective, the urban sustainability level in the north of Anhui is clearly lower than that in the southern and central regions of Anhui. This result is consistent with the urban development of Anhui province. Hefei is the capital city of Anhui province, a large amount of funds have been invested in developing the city’s economic, social, and ecological constructions and great achievements were made in the recent years, so it is substantially better than other cities in reality. As a coal resource-based city, the economic and social developments of Huainan are slow and its ecological damage is more serious than other cities, so its sustainability level is the lowest. 

Next, the numerical calculations and comparative analysis are carried out from economy, society, and ecology.

#### 5.3.1. Economic Sustainability

**Figure 4:**
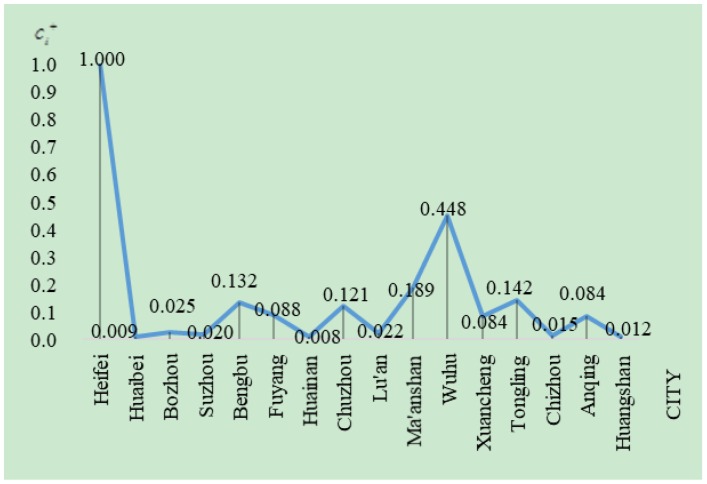
The comparison of economic sustainability of cities in Anhui province.

Figure 4 shows the economic dimensional index data of each city. In the economic dimension, Hefei City, as the only second-tier city in Anhui province, has a significantly higher economic level than other cities, and the overall economic level of the northern cities of Anhui is obviously less than that of the southern and central cities. By comparing the various economic development indices, we can find that it is necessary to improve the overall economic level in order to enhance the urban sustainability, and economic growth should maintain steady. If growth is too fast, the quality of economic development is difficult to guarantee. On the other hand, if growth is too slow it will limit the urban sustainable development. For economically developed cities, such as Hefei and Wuhu, the policy-maker should aim to accomplish the optimization of urban industrial structure and moderate economic growth. In the future, the policy-maker should increase the investment in emerging industries, such as the industries of new-energy vehicles, robotics, modern agriculture machinery equipment, and so on; apply technological innovation for driving industrial upgrading; and accelerate the development of the tertiary industry and modern agriculture. 

#### 5.3.2. Social Sustainability

**Figure 5:**
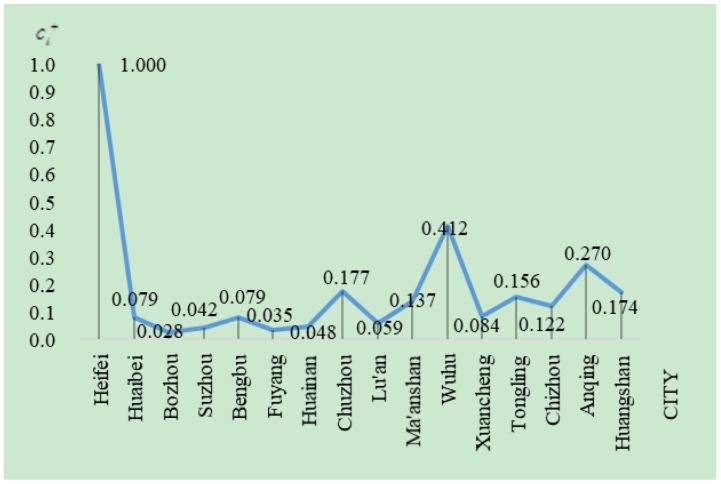
The comparison of social sustainability of cities in Anhui province.

Figure 5 shows the urban sustainability level from the social dimension. It indicates that the social indices of central cities and their surrounding cities are higher than those of other cities. Comparing the various social indices, we find that urban development should focus on the improvement of the industrial structure, scientific regulation, and active market intervention, which can promote employment and improve the life quality of residents. Furthermore, when the city is developing, it is also necessary to strengthen the city’s security measures and legal system, cultivate residents’ legal awareness, and enhance urban security. Cities with insufficient scientific and educational indices should strengthen their construction of urban innovation capabilities, and pay more attention to the popularization of basic cultural education and university construction. Anhui is an undeveloped and densely populated province; therefore, its social development is slow. Although the social development levels of Hefei city and Wuhu city are higher than other cities, they are lower than many cities in other provinces. Therefore, policy-makers need to develop measures to enhance population quality, improve urban life quality, and social welfare. In addition, more investment in education and culture should be taken in the future, especially in the northern cities of Anhui province.

#### 5.3.3. Ecological Sustainability

**Figure 6:**
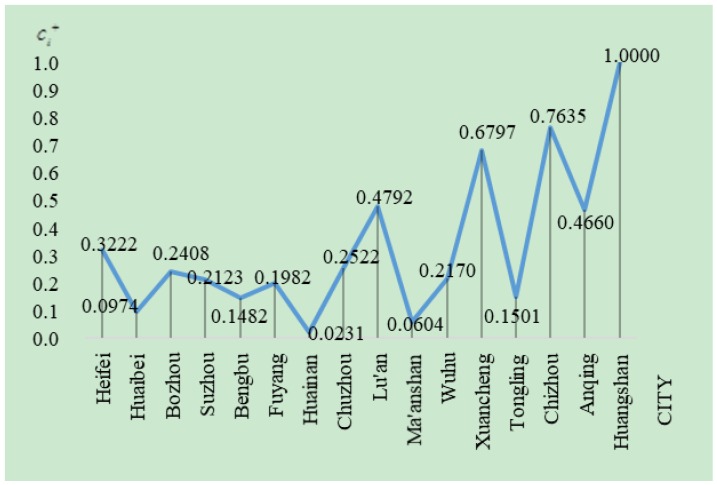
The comparison of ecological sustainability of cities in Anhui province.

Figure 6 shows the evaluation results of the ecological indices. It is known that Huangshan, as a national 5A-level tourist scenic spot with world cultural and natural heritage, has a leading position among the cities of Anhui province in terms of ecological indices. Moreover, the ecological sustainability levels of the cities in the mountainous areas of southern Anhui are obviously higher than those of other cities. Most southern cities are ecotourism cities: they possess many rich tourism resources so they can focus their tertiary industry around tourism. In addition, tourism needs the support of hotel industry, catering industry, and transportation. Therefore, the stakeholders also need to accelerate the coordinated development of related industries. For example, adjusting hotel structure to develop starred hotel, constructing high-speed rail, and so on. On the other hand, some cities with abundant mineral resources and a developed heavy industry, such as Huaibei, Huainan, Tongling, and Ma’anshan, consume more petroleum and mineral energy. In addition, the lower productive efficiency will also result in serious pollution. Therefore, the ecological sustainability of these cities is at an obviously low level. Especially, the location of Tongling is similar to Anqing and Xuancheng, but its ecological sustainability level is lower than them. Hence, we can infer that the heavy industry in cities has a significant negative impact on ecological sustainability. These cities need to attach importance to the innovation and development of energy-saving, environmental protection, and bioenergy technologies, and implement rational planning and adjustments for the layout of the secondary industries to reduce the output of by-products that have negative effects on the ecosystem, and further realize green industrial production with high technology, high efficiency, low pollution, and low consumption.

## 6. Conclusions

In this study, we present a modified TOPSIS based on grey relational analysis to evaluate the urban sustainability. The main contributions of this paper can be summarized as follows. (1) Based on the existing urban sustainability theory and the concept of urban sustainability, an evaluation index system for urban sustainability is established with consideration of the total quantity, quality, and efficiency of economic, social, and ecological development. This evaluation index system is human-centered and can provide a comprehensive evaluation for urban sustainable development. (2) One modified evaluation method of urban sustainability is proposed to rank the sustainability level of cities. In this method, the entropy method is used to determine the weight of each index based on its evaluation score and the grey relation analysis is used to decrease the uncertainty existing in the evaluation process. (3) A case concerning the sustainability evaluation of 16 cities in Anhui province is presented. The results show that the sustainability of Hefei is the best of 16 cities, and its sustainable economic and social development are both better than the others. However, with respect to ecological dimensions, Huangshan city has the highest sustainability level among them. These results can provide related agents with suggestions and decision supports for urban sustainable development of Anhui province.

The evaluation method in this paper fully uses the objective statistical data of the economy, society, and environment, but it does not consider their interactions. Generally, the economic, social, and ecological development of one city interacts with each other. Therefore, we will continue to improve the evaluation method considering the interactions of economic, social, and ecological sustainable development in the future.

## Figures and Tables

**Table 1:** Evaluation index system of urban sustainability.

Dimension	Criteria	Sub-criteria	Code
Economic Indices	Economic aggregate	GDP (100 million RMB)	EC1
		Investment in fixed assets (100 million RMB)	EC2
		Retail sales of consumer goods (100 million RMB)	EC3
		Total import and export volume (100 million dollar)	EC4
	Economic quality	GDP per capita (RMB)	EC5
		Disposable income of urban residents per capita (RMB)	EC6
		Disposable income of rural residents per capita (RMB)	EC7
		Output value of high-tech industries (100 million RMB)	EC8
	Economic increment	GDP growth rate (%)	EC9
		Growth rate of total investments with respect to fixed assets (%)	EC10
		Growth rate of consumer goods’ retail sales (%)	EC11
		Growth rate of the Total import and export volume (%)	EC12
Social Indices	Population	Natural growth rate of the population (‰)	SC13
		Population density (Person/KM^2^)	SC14
	Urban construction	Park land per capita (m^2^)	SC15
		Per capita living area in urban areas (m^2^)	SC16
		per capita road area (m^2^)	SC17
	Quality of life	Urban Engel coefficient (%)	SC18
		Rural Engel coefficient (%)	SC19
		Urban unemployment rate (%)	SC20
		Percentage of Urban residents with basic medical insurance (%)	SC21
		Number of practicing physicians per 10,000 people (Persons)	SC22
		Number of criminal cases (Pieces)	SC23
	Science, education and culture	Number of library collections per million people (10 thousand copies)	SC24
		Patent applications (Pieces)	SC25
		Years of education per capita (Years)	SC26
		Number of graduates from ordinary colleges and universities (Persons)	SC27
Ecological Indices	Natural resources	Water resources per capita (m^3^/Persons)	EL28
		Agricultural land area (1000 ha)	EL29
		Forest coverage rate (%)	EL30
	Environmental pollution	Total amount of industrial waste gas discharge (100 million Nm^3^)	EL31
		Total amount of industrial wastewater discharge (10 thousand Ton)	EL32
		Amount of industrial solid waste (10 thousand Ton)	EL33
		Urban sewage discharge (10 thousand m^3^)	EL34
		Proportion of air quality equal to and better than level II (%)	EL35
	Ecological construction	Treatment rate of industrial effluents (%)	EL36
		Financial expenditures on energy conservation and environmental protection (10 thousand RMB)	EL37
		Green coverage rate of built-up areas (%)	EL38
		Comprehensive utilization rate of industrial solid wastes (%)	EL39

**Table 2:** Evaluation index data of eight cities of Anhui province in the north of Huaihe River.

Index	Heifei	Huaibei	Bozhou	Suzhou	Bengbu	Fuyang	Huainan	Chuzhou
EC1	6274.4	799.0	1046.1	1351.8	1385.8	1401.9	963.8	1422.8
EC2	6501.2	958.9	874.9	1270.0	1666.4	1292.6	955.0	1699.2
EC3	2445.7	315.9	492.1	476.9	643.9	759.4	512.5	515.2
EC4	203.31	5.77	5.02	7.59	23.41	14.96	3.32	20.46
EC5	80,138	36,427	20,611	24,270	41,855	17,642	27,990	35,302
EC6	34,852	27,248	25,053	25,533	28,653	25,483	28,098	26,286
EC7	17,059	10,653	10,576	9917	12591	9776	10,848	10,956
EC8	5899.8	477.9	363.9	233.3	1251.4	461.3	110.9	1424.9
EC9	9.8	5.0	8.9	9.1	9.4	9.0	6.6	9.2
EC10	11.1	3.6	14.0	12.1	14.3	28.6	3.8	16.6
EC11	12.0	11.5	12.8	12.3	12.9	12.5	11.6	12.7
EC12	−8.1	6.3	3.6	−38.2	−24.8	−17.1	14.1	−14.6
SC13	5.98	8.39	10.43	9.27	10.03	11.02	6.82	6.26
SC14	3553	2550	3936	3484	2618	2353	2247	1587
SC15	13.50	16.72	13.39	13.41	13.03	13.95	12.60	14.48
SC16	35.75	41.00	50.00	34.55	35.40	52.00	31.30	36.39
SC17	17.04	16.91	45.53	27.90	20.12	26.22	15.43	43.90
SC18	33.00	31.28	30.30	22.63	36.80	32.40	35.60	31.80
SC19	36.30	31.70	34.80	26.48	36.40	33.50	38.40	39.00
SC20	3.03	4.16	2.76	2.42	2.86	2.68	4.00	3.22
SC21	50.23	43.67	18.76	43.81	29.29	1.68	47.59	45.32
SC22	26.42	19.78	8.75	13.30	17.02	12.42	16.06	13.90
SC23	61,179	7425	11,009	18,616	14,431	20,005	10,453	17,288
SC24	72.02	42.44	14.87	14.43	33.91	5.49	16.06	23.26
SC25	50,792	2604	3438	3324	9402	8510	5540	12,628
SC26	10.89	9.54	8.29	8.66	9.62	8.41	9.37	8.97
SC27	135,934	9446	4449	5487	15,911	8975	17,393	12,978
EL28	1101	344	532	500	656	490	588	1577
EL29	821.5	201.1	690.1	768.7	449.6	771.8	414.0	1069.1
EL30	14.03	19.18	17.78	25.56	16.74	18.63	9.56	16.77
EL31	1902.9	1335.0	394.2	785.8	504.7	678.7	2628.7	975.9
EL32	5129.8	3661.1	1878.6	3572.5	1917.0	2500.2	4081.8	3799.0
EL33	992.0	1174.3	280.1	299.8	175.6	604.3	2779.6	127.7
EL34	50,342	4738	5379	4100	15,370	7128	7227	6052
EL35	69.10	66.10	70.80	62.60	67.80	66.40	74.90	65.80
EL36	99.71	97.97	94.09	98.05	99.51	94.09	97.47	96.71
EL37	223,958	66,546	109,505	81,951	54,556	90,182	51,102	68,142
EL38	41.78	44.98	36.79	42.66	40.02	38.55	40.8	41.48
EL39	73.65	95.98	97.15	87.52	98.42	95.15	76.79	77.31

**Table 3:** Evaluation index data of eight cities of Anhui province in the south of Huaihe River.

Index	Lu’an	Ma’anshan	Wuhu	Xuancheng	Tongling	Chizhou	Anqing	Huangshan
EC1	1108.2	1493.8	2699.4	1057.8	957.3	589.0	1531.2	576.8
EC2	1075.0	2064.6	3006.9	1414.3	1196.9	652.6	1521.9	597.7
EC3	541.5	470.6	828.2	475.8	305.7	222.1	681.7	313.1
EC4	6.17	29.55	68.19	18.52	45.81	5.20	24.45	6.35
EC5	23,298	65,833	73,715	40,740	59,960	40,919	33,294	41,905
EC6	24,728	38,142	32,315	30,877	30,633	26,261	26,502	28,393
EC7	9960	17,719	17,307	13,379	12,054	12,409	10,814	12,869
EC8	381.2	1031.6	3748.4	756.3	956.1	334.4	590.1	198.0
EC9	7.2	9.0	9.7	8.7	9.1	8.1	8.0	7.8
EC10	8.2	11.0	11.0	10.2	12.6	8.7	9.8	8.2
EC11	11.7	12.4	13.0	12.6	12.3	12.2	12.0	11.4
EC12	7.7	−16.3	−16.3	−18.6	1.4	19.0	−28.2	2.9
SC13	5.71	5.04	3.56	2.84	4.09	3.85	5.32	2.64
SC14	3630	4205	1882	2710	2513	1204	2277	835
SC15	14.84	14.98	13.42	14.10	17.67	17.08	13.96	14.88
SC16	37.89	34.00	35.00	39.01	36.30	42.20	45.60	48.40
SC17	24.24	19.14	26.34	30.91	12.09	25.65	18.69	22.78
SC18	36.10	34.90	35.20	32.60	32.80	33.10	35.60	34.00
SC19	36.90	32.50	38.60	35.00	35.50	32.12	38.70	34.20
SC20	4.00	2.93	3.39	3.06	3.09	3.29	3.22	3.68
SC21	49.55	44.24	51.69	25.79	79.42	20.83	46.64	42.33
SC22	15.20	19.19	20.49	18.37	19.40	17.73	15.29	21.91
SC23	10,768	9391	16282	7359	7123	4928	11,524	4295
SC24	12.18	53.72	55.19	32.06	63.80	31.97	105.50	70.48
SC25	8779	9477	26,680	5261	3480	4099	17,022	1516
SC26	8.77	8.99	10.11	8.59	9.10	9.06	8.91	8.84
SC27	11,745	14,564	34,685	1955	10,211	5640	12,887	5765
EL28	3162	1745	1938	7098	2079	8638	4154	11,500
EL29	1283.3	299.7	443.1	1079.0	190.1	724.9	1035.8	903.4
EL30	45.67	18.63	21.20	59.12	25.95	61.64	40.90	83.25
EL31	455.4	6056.2	2960.8	1360.5	30,24.1	1101.9	1147.6	54.4
EL32	809.8	7557.7	3302.1	1810.7	3934.9	945.2	4017.8	706.7
EL33	968.9	2280.2	302.1	469.2	1854.9	139.5	185.7	18.7
EL34	4806	9322	14,900	2194	5289	2804	6432	3167
EL35	81.40	74.30	80.30	81.60	77.30	79.30	73.40	97.30
EL36	98.42	99.64	93.56	93.94	93.10	93.90	97.37	94.54
EL37	86,276	54,491	74,256	68,117	47,705	75,743	69,692	80,064
EL38	41.46	44.10	40.58	41.50	48.50	43.12	43.10	46.67
EL39	40.07	91.02	91.62	77.15	92.37	88.34	97.33	79.50

**Table 4:** The weight of each index using the entropy method.

Index	$E_{j}$	$\omega_{j}$	Index	$E_{j}$	$\omega_{j}$	Index	$E_{j}$	$\omega_{j}$
EC1	0.787	0.047	SC14	0.926	0.016	SC27	0.700	0.066
EC2	0.802	0.043	SC15	0.897	0.022	EL28	0.749	0.055
EC3	0.824	0.039	SC16	0.904	0.021	EL29	0.907	0.020
EC4	0.669	0.073	SC17	0.906	0.021	EL30	0.851	0.033
EC5	0.889	0.024	SC18	0.885	0.025	EL31	0.970	0.007
EC6	0.851	0.033	SC19	0.878	0.027	EL32	0.962	0.008
EC7	0.834	0.036	SC20	0.933	0.015	EL33	0.956	0.010
EC8	0.741	0.057	SC21	0.954	0.010	EL34	0.975	0.005
SC9	0.965	0.008	SC22	0.949	0.011	EL35	0.905	0.021
SC10	0.910	0.020	SC23	0.975	0.005	EL36	0.890	0.024
SC11	0.923	0.017	SC24	0.889	0.024	EL37	0.825	0.038
SC12	0.940	0.013	SC25	0.782	0.048	EL38	0.944	0.012
SC13	0.924	0.017	SC26	0.899	0.022	EL39	0.971	0.06

**Table 5:** Positive and negative ideal solutions for each index.

Index	Positive Solution	Negative Solution	Index	Positive Solution	Negative Solution	Index	Positive Solution	Negative Solution
EC1	0.036	0.003	SC14	0.001	0.006	SC27	0.061	0.001
EC2	0.033	0.003	SC15	0.007	0.005	EL28	0.036	0.001
EC3	0.030	0.003	SC16	0.007	0.004	EL29	0.008	0.001
EC4	0.065	0.001	SC17	0.009	0.002	EL30	0.018	0.002
EC5	0.011	0.002	SC18	0.004	0.007	EL31	0.000	0.005
EC6	0.011	0.007	SC19	0.005	0.007	EL32	0.000	0.004
EC7	0.013	0.007	SC20	0.003	0.005	EL33	0.000	0.006
EC8	0.045	0.001	SC21	0.005	0.000	EL34	0.000	0.005
EC9	0.002	0.001	SC22	0.004	0.001	EL35	0.007	0.004
EC10	0.011	0.001	SC23	0.000	0.004	EL36	0.006	0.006
EC11	0.005	0.004	SC24	0.013	0.001	EL37	0.024	0.005
EC12	0.004	−0.007	SC25	0.038	0.001	EL38	0.004	0.003
SC13	0.002	0.007	SC26	0.007	0.005	EL39	0.002	0.001

**Table 6:** Distance between the sustainability of each city and the positive and negative ideal solutions.

City	${L_{i}}^{+}$	${L_{i}}^{-}$	City	${L_{i}}^{+}$	${L_{i}}^{-}$
Heifei	1.000	0.000	Lu’an	0.071	0.931
Huaibei	0.000	0.998	Ma’anshan	0.144	0.924
Bozhou	0.001	0.973	Wuhu	0.443	0.671
Suzhou	0.007	1.000	Xuancheng	0.106	0.856
Bengbu	0.125	0.944	Tongling	0.125	0.915
Fuyang	0.062	0.953	Chizhou	0.052	0.817
Huainan	0.011	0.991	Anqing	0.167	0.868
Chuzhou	0.138	0.916	Huangshan	0.055	0.727

**Table 7:** Grey relational degree of each city.

City	${Q_{i}}^{+}$	${Q_{i}}^{-}$	City	${Q_{i}}^{+}$	${Q_{i}}^{-}$
Heifei	1.000	0.000	Lu’an	0.135	0.779
Huaibei	0.053	0.944	Ma’anshan	0.123	0.730
Bozhou	0.099	0.894	Wuhu	0.333	0.365
Suzhou	0.063	0.918	Xuancheng	0.226	0.662
Bengbu	0.098	0.768	Tongling	0.148	0.741
Fuyang	0.101	0.828	Chizhou	0.258	0.716
Huainan	0.000	1.000	Anqing	0.206	0.624
Chuzhou	0.167	0.683	Huangshan	0.370	0.648

**Table 8:** The relative closeness and ranking of cities.

City	${S_{i}}^{+}$	${S_{i}}^{-}$	${c_{i}}^{+}$	Rank	City	${S_{i}}^{+}$	${S_{i}}^{-}$	${c_{i}}^{+}$	Rank
Heifei	1.0000	0.0000	1.0000	1	Ma’anshan	0.1335	0.8272	0.1390	9
Wuhu	0.3877	0.5183	0.4279	2	Bengbu	0.1114	0.8558	0.1152	10
Huangshan	0.2128	0.6875	0.2364	3	Lu’an	0.1031	0.8552	0.1076	11
Anqing	0.1866	0.7464	0.2000	4	Fuyang	0.0817	0.8908	0.0840	12
Xuancheng	0.1662	0.7588	0.1797	5	Bozhou	0.0497	0.9333	0.0505	13
Chizhou	0.1550	0.7669	0.1681	6	Suzhou	0.0349	0.9589	0.0351	14
Chuzhou	0.1525	0.7996	0.1602	7	Huaibei	0.0267	0.9713	0.0267	15
Tongling	0.1363	0.8279	0.1414	8	Huainan	0.0056	0.9955	0.0056	16
